# Evaluation of the antitumor effect of lectin obtained from the latex of *Euphorbia tirucalli *against tumor cells of Ehrlich

**DOI:** 10.1186/1753-6561-8-S4-P38

**Published:** 2014-10-01

**Authors:** Aline Richter, Caroline Mota, Fernanda Santiago, Marcelo Barbosa

**Affiliations:** 1 Universidade Federal de Uberlândia, Uberlândia, Brazil

## Introduction

According to the World Health Organization, cancer is the second leading cause of worldwide death and breast cancer is among the most frequent types. Conventional therapies show many side effects, high cost and sometimes are inefficient in the total eradication of tumors. Looking for new alternatives to cure this disease a lot of studies have been developed in the field of medicinal plants, due to the vast distribution and use of folk medicine by patients as a single treatment or as a supplement to conventional treatment, obtaining positive effects in several cases. Currently, research has been directed for the characterization and purification of molecules and substances in order to identify the responsible components for therapeutic activity and minimizing side effects. *Euphorbia tirucalli *popularly known as Labyrinth, is a frequently cited plant in traditional medicine for its use as an antitumor agent. The present study aims to purify the lectin fraction obtained from the latex of *E. tirucalli*, and verify the relation and contribution of this bioactive substance, as well as the nonbonding fractions and crude extract, the activity against tumor Erhlich.

## Methods

We performed tests of hemagglutination and hemagglutination inhibition with different carbohydrates to the identification of lectin activity. After confirmation of binding sites for sugar D- galactose, purifications were performed by affinity chromatography and biological assays with the fraction ligand and the void as well with the crude extract of the latex for stimulation of macrophages in culture cytotoxicity assay, and evaluation of the effect of the fractions in the evolution of the tumor (breast adenocarcinoma) in Balb C by weight variation of animals.

## Results

The results of the experiments show that the crude extract of *E. tirucalli *components has cytotoxicfeatures that promote the lysis of red blood cells in high concentrations, however, at higher dilutions was identified lectin activity. From the SDS -PAGE, we observed the protein profile of the crude extract and purification efficiency of affinity column fraction lectin( binder ). Regarding lectin's feature, this was evidenced in hemagglutination tests subsequent to the exclusion chromatograms of the hemolytic effect. MTT assay in front of tumor cells, the fraction lectin have not shown a cytotoxic effect, however, when carried immunofluorescence slides, it was found that the fraction of *E. tirucalli *interact with receptors present on the surface of tumor cells and lymphocytes.

## Conclusion

The chromatographic method was efficient in the purification process of the fraction with lectin activity from the latex of *E. tirucalli *eliminating the cytotoxic effects attributed to the crude extract. From this separation was observed the interaction of fraction lectin with receptors surfaceof tumor cells which could interfere with the tumor microenvironment. Although the production of TNF - α by macrophages in test stimulation may be related to the antitumor activity and suggest a mechanism of action for this biomolecule by inducing inflammatory response.
